# Supplementary material for: Histone binding of ASF1 is required for fruiting body development but not for genome stability in the filamentous fungus Sordaria macrospora
Source: mBio. 2023 Dec 19;15(1):e02896-23. doi: 10.1128/mbio.02896-23 (PMC10790691; doi:10.1128/mbio.02896-23)
Supplement: Supplemental Figures and Tables — Tables S1-S5 and S7 and Figures S1-S11. [file mbio.02896-23-s0001.pdf]

Supplemental data for manuscript "Histone binding of ASF1 is required for fruiting body development, but not for genome stability in the filamentous fungus *Sordaria macrospora*"

This document contains the following supplemental data:

Supplemental Tables 1-5 and 7

Supplemental Figures 1-11

Supplemental Table 6 is provided separately as an Excel file.

Supplemental Table 1. *S. macrospora* strains used in this study.

Name of strain	Genotype	Comments
SN1693	Wild type	Reference strain
SN1891	Fus1-1	Spore color mutant
SN1983	Δasf1::*hph*; sterile	*asf1* deletion mutant
SJM 25.4.2	Δasf1::*hph* + pDS23-ASF1-WT	*asf1* complementation strain with wild type gene
SJM 26.8.5	Δasf1::*hph* + pDS23-ASF1-D37A	*asf1* complementation strain with D37A substitution
SJM 27.4.1	Δasf1::*hph* + pDS23-ASF1-V94R	*asf1* complementation strain with V94R substitution
JB 41.1.1	Δasf1::*hph* + pGG-ASF1-152T	*asf1* complementation strain with truncation in pos. 152
JB 42.1.2	Δasf1::*hph* + pGG-ASF1-185T	*asf1* complementation strain with truncation in pos. 185
JB 51.1.13	Δasf1::*hph* + pGG-ASF1-210T	*asf1* complementation strain with truncation in pos. 210
SJM 44.1	wild type + pGG_H3_Flag + pDS23_asf1_WT	Performance of CoIP experiments with ASF1-eGFP wild type construct and H3-Flag
SJM 45.1	wild type + pGG_H3_Flag + pDS23_asf1_D37A	Performance of CoIP experiments with ASF1-eGFP D37A construct and H3-Flag
SJM 35.8	wild type + pGG_H3_Flag + pDS23_asf1_V94R	Performance of CoIP experiments with ASF1-eGFP V94R construct and H3-Flag
SJM 47.1	wild type + pGG_H3_Flag + pDS23_eGFP	Performance of CoIP experiments with eGFP construct and H3-Flag
SJM 48.5	wild type + pGG_H4_Flag + pDS23_asf1_WT	Performance of CoIP experiments with ASF1-eGFP wild type construct and H4-Flag
SJM 49.2	wild type + pGG_H4_Flag + pDS23_asf1_D37A	Performance of CoIP experiments with ASF1-eGFP D37A construct and H4-Flag
SJM 50.2	wild type + pGG_H4_Flag + pDS23_asf1_V94R	Performance of CoIP experiments with ASF1-eGFP V94R construct and H4-Flag
SJM 51.1	wild type + pGG_H4_Flag + pDS23_eGFP	Performance of CoIP experiments with eGFP construct and H4-Flag
JB 25.2	wild type + pGG_H3_Flag + pGG-ASF1-152T	Performance of CoIP experiments with ASF1-eGFP 152T construct and H3-Flag
JB 35.2	wild type + pGG_H3_Flag + pGG-ASF1-185T	Performance of CoIP experiments with ASF1-eGFP 185T construct and H3-Flag
JB 52.2	wild type + pGG_H3_Flag + pGG-ASF1-210T	Performance of CoIP experiments with ASF1-eGFP 210T construct and H3-Flag
JB 34.3	wild type + pGG_H4_Flag + pGG-ASF1-152T	Performance of CoIP experiments with ASF1-eGFP 152T construct and H4-Flag
JB 36.5	wild type + pGG_H4_Flag + pGG-ASF1-185T	Performance of CoIP experiments with ASF1-eGFP 185T construct and H4-Flag
JB 53.2	wild type + pGG_H4_Flag + pGG-ASF1-210T	Performance of CoIP experiments with ASF1-eGFP 210T construct and H4-Flag

Supplemental Table 2. Oligonucleotides used in this study.

Primer	Sequence 5'-3'	remarks
asf1_1	TCATCGCAGCTTGACTAACAGCTACATGTCTGTC GTTTCGCTTCTCGGGG	*asf1* from start downstream with 5' overlap with plasmid pDS23
asf1_2	ACAGCTCCTCGCCCTTGCTCACCATTTGAGCCAT GGCCATACCCTGCGGT	*asf1* from end upstream with 5' overlap with plasmid pDS23
asf1_3	TGGGCGTCACGAGGATTTTGTTGACGTGCGC	for mutagenesis of *asf1* V94R
asf1_9	CGTCAACAAAATCCTCGTGACGCCCAGAAGCTCG	for mutagenesis of *asf1* V94R
asf1_5	GTTCGCGTACCAGCCCTCGAGTGGAAGCTCA	for mutagenesis of *asf1* D37A
asf1_6	TCCACTCGAGGGCTGGTACGCGAACGCGACG	for mutagenesis of *asf1* D37A
GG-ASF1-152T-FW	GGCTACGGTCTCGTGGTATGTCTGTCGTTTCGCTTC	for amplification of *asf1* 152T
GG-ASF1-152T-RV	GGCTACGGTCTCAATCCCTTGATGGCAAAGCGCGTAAC	for amplification of *asf1* 152T
GG-ASF1-185T-FW	GGCTACGGTCTCCTGGTATGTCTGTCGTTTCGCTTCTCG	for amplification of *asf1* 185T
GG-ASF1-185T-RV	GGCTACGGTCTCTATCCGGCAAGTTCATCGGCGCC	for amplification of *asf1* 185T
GG-ASF1-210T-FW	GGCTACGGTCTCTTGGTATGTCTGTCGTTTCGCTTCTC	for amplification of *asf1* 210T
GG-ASF1-210T-RV	GGCTACGGTCTCCATCCGACGATGGCGCCGTTCAATTG	for amplification of *asf1* 210T
asf1_veri_fw	TCAATCTTGTCGCTAACCG	verification of *asf1* presence in PCR tests
asf1_veri_rv	GTCAACACTGCCATCCTC	verification of *asf1* presence in PCR tests
egfp_rv	ACTTGTGGCCGTTTACGTCG	verification of *asf1* presence in PCR tests by binding to the eGFP tag of complementation vectors
1751	GCCATATTTTCCTGCTCTCC	verification of *asf1* presence in PCR tests by binding to the gpd promotor of complementation vectors
asf1_dupl1	CTCACCAGACCTGAAGTTGCGTCC	verification of duplication in Δasf1 strains
asf1_dupl2	TAGAGGTACACTACGCCCATCTCC	verification of duplication in Δasf1 strains
asf1_dupl3	TCAGCGATCATTTGTCAGTGTCCG	verification of duplication in Δasf1 strains
asf1_dupl4	GTGGGTGGAAAGTCAAAAGTCTGG	verification of duplication in Δasf1 strains
asf1_dupl5	ATATAGCTGGTGATCCCTTCCAGC	verification of deletion in duplication in Δasf1 strains
asf1_dupl6	ATGGGGAGATTACACAGGTGTTCG	verification of deletion in duplication in Δasf1 strains

Supplemental Table 3. Plasmids used in this study.

Plasmid	Characteristics	Comments
pDS23	8,8 kb, P*gpd::eGFP::*TrpC, *ura, amp, lacZ*	eGFP control for CoIP experiments, cloning basis for complementation vectors, eGFP tag
pDS23_asf1_WT	9,9 kb, pDS23-derivate, asf1(WT)-*egfp*	*asf1* wild type complementation and CoIP vector, eGFP tag
pDS23_asf1_D37A	9,9 kb, pDS23-derivate, *asf1*(D37A)-*egfp*	*asf1* D37A complementation and CoIP vector, eGFP tag
pDS23_asf1_V94R	9,9 kb, pDS23-derivate, *asf1*(V94R)-*egfp*	*asf1* V94R complementation and CoIP vector, eGFP tag
pGG-H3-Flag	5,8 kb, P*trpC*::*Nat*, P*gpd*, 3xFlag, *LacZa*, T*rpC*, *amp*, pUC-ori, *SMAC_02363* (histone H3)	H3 CoIP vector, FLAG tag
pGG-H4-Flag	5,8 kb, P*trpC*::*Nat*, P*gpd*, 3xFlag, *LacZa*, T*rpC*, *amp*, pUC-ori,*SMAC_02364* (histone H4)	H4 CoIP vector, FLAG tag
pGG-ASF1-152T	6,6 kb, P*trpC*::*Nat*, P*gpd*, eGFP, *LacZa*, T*rpC*, *amp*, pUC-ori, *asf1*(152T)-*egfp*	*asf1* 152T complementation and CoIP vector, eGFP tag
pGG-ASF1-185T	6,8 kb, P*trpC*::*Nat*, P*gpd*, eGFP, *LacZa*, T*rpC*, *amp*, pUC-ori, *asf1*(185T)-*egfp*	*asf1* 185T complementation and CoIP vector, eGFP tag
pGG-ASF1-210T	6,9 kb, P*trpC*::*Nat*, P*gpd*, eGFP, *LacZa*, T*rpC*, *amp*, pUC-ori, *asf1*(210T)-*egfp*	*asf1* 210T complementation and CoIP vector, eGFP tag

Supplemental table 4. Western Blot protocols used in this study.

**H3K27me3 detection**	**H3K9me detection**	**H3K56ac detection**
4 h blocking in TBST (0,1 % Tween-20) with 5 % (w/v) non-fat dry milk<br><br>10 min wash in TBST (0,1 % Tween-20)<br><br>16 h incubation Anti H3K27Me3 (Cell Signaling) 1:1000 in TBST (0,1 % Tween-20) with 5 % (w/v) non-fat dry milk<br><br>3 x 10 min wash in TBST (0,1 % Tween-20)<br><br>1 h incubation Anti-Rabbit IgG HRP linked (Cell Signaling) 1:5000 in TBST (0,1 % Tween-20) with 5 % (w/v) non-fat dry milk<br><br>3 x 10 min wash in TBST (0,1 % Tween-20)	4 h blocking in TBST (0,1 % Tween-20) with 5 % (w/v) non-fat dry milk<br><br>10 min wash in TBST (0,1 % Tween-20)<br><br>16 h incubation Anti H3K9Me (Merck Millipore) 1:1000 in TBST (0,1 % Tween-20) with 5 % (w/v) non-fat dry milk<br><br>3 x 10 min wash in TBST (0,1 % Tween-20)<br><br>1 h incubation Anti-Rabbit IgG HRP linked (Cell Signaling) 1:5000 in TBST (0,1 % Tween-20) with 5 % (w/v) non-fat dry milk<br><br>3 x 10 min wash in TBST (0,1 % Tween-20)	4 h blocking in TBST (0,1 % Tween-20) with 1 % (w/v) BSA<br><br>16 h incubation Anti H3K56Ac (Active Motif) 1:1000 in TBST (0,1 % Tween-20) with 1 % (w/v) BSA<br><br>10 min wash in TBST (0,1 % Tween-20)<br><br>2 h incubation Anti-Rabbit IgG HRP linked (Cell Signaling) 1:5000 in TBST (0,1 % Tween-20) with 1 % (w/v) BSA<br><br>3 x 10 min wash in TBST (0,1 % Tween-20)
**H3K9Ac**	**eGFP tag detection**	**FLAG tag detection**
4 h blocking in TBST (0,1 % Tween-20) with 1 % (w/v) BSA<br><br>16 h incubation Anti H3K9Ac (Active Motif) 1:1000 in TBST (0,1 % Tween-20) with 1 % (w/v) BSA<br><br>10 min wash in TBST (0,1 % Tween-20)<br><br>2 h incubation Anti-Rabbit IgG HRP linked (Cell Signaling) 1:5000 in TBST (0,1 % Tween-20) with 1 % (w/v) BSA<br><br>10 min wash in TBST (0,1 % Tween-20)	1 h blocking in PBST (0,1 % Tween-20) with 5 % (w/v) non-fat dry milk<br><br>10 min wash in PBS<br><br>1h incubation in living colors JL-8 (Clontech) 1:2000 in PBST (0,1 % Tween-20) with 5 % (w/v) non-fat dry milk<br><br>2 x 5 min wash in PBST (0,1 % Tween-20)<br><br>1 h incubation Anti-Mouse IgG HRP linked (Cell Signaling) 1:1000 in PBST (0,1 % Tween-20) with 5 % (w/v) non-fat dry milk<br><br>4 x 10 min wash in PBST (0,1 % Tween-20)	1 h blocking in TBST (0,05 % Tween-20) with 5 % (w/v) non-fat dry milk<br><br>10 min wash in TBS<br><br>1h incubation Mouse Anti-FLAG M2 (Sigma-Aldrich) 1:2000 in TBST (0,05 % Tween-20) with 5 % (w/v) non-fat dry milk<br><br>10 min wash TBS<br><br>1 h incubation Anti-Mouse IgG HRP linked (Cell Signaling) 1:5000 in TBST (0,05 % Tween-20) with 5 % (w/v) non-fat dry milk<br><br>3 x 10 min wash in TBST (0,05 % Tween-20)

Supplemental table 5. Hi-C ligation events.

Strain	Genotype	Replicate	Obtained reads	Mapped pairs	HI-C contacts	% of reads in HI-C contacts
SN1693	wild type young	1	50280915	46429907	4191907	8.33
SN1693	wild type young	2	55234668	51511005	10427734	18.87
SN1693	wild type old	1	48173321	43876915	2534375	5.26
SN1693	wild type old	2	149315718	135385996	18809722	12.59
SN1983	Δasf1	1	50056244	45904670	5244496	10.47
SN1983	Δasf1	2	55780155	50555623	8609712	15.43

Supplemental table 7. Strains tested for the duplication on chromosome 2.

Name of strain	Genotype at the *asf1* gene locus	Duplication on chromosome 2	Fertility and phenotype
S 689	wild type	not present	fertile, wild type phenotype
S 690	wild type	present	fertile, wild type phenotype
S 692	wild type	present	fertile, wild type phenotype
S 693	wild type	present	fertile, wild type phenotype
S 694	wild type	not present	fertile, wild type phenotype
S 695	wild type	not present	fertile, wild type phenotype
S 696	wild type	not present	fertile, wild type phenotype
S 697	wild type	not present	fertile, wild type phenotype
S 698	wild type	not present	fertile, wild type phenotype
S 699	wild type	present	fertile, wild type phenotype
S 700	wild type	present	fertile, wild type phenotype
S 701	wild type	not present	fertile, wild type phenotype
S 703	wild type	present	fertile, wild type phenotype
S 704	wild type	not present	fertile, wild type phenotype
S 705	wild type	not present	fertile, wild type phenotype
S 706	wild type	not present	fertile, wild type phenotype
S 707	wild type	not present	fertile, wild type phenotype
S 708	wild type	not present	fertile, wild type phenotype
S 709	wild type	not present	fertile, wild type phenotype
S 710	wild type	present	fertile, wild type phenotype
S 896	wild type	not present	fertile, wild type phenotype
S 897	wild type	present	fertile, wild type phenotype
S 898	wild type	not present	fertile, wild type phenotype
S 899	wild type	not present	fertile, wild type phenotype
S 900	wild type	not present	fertile, wild type phenotype
S 901	wild type	not present	fertile, wild type phenotype
S 902	wild type	not present	fertile, wild type phenotype
S 903	wild type	not present	fertile, wild type phenotype
S 904	wild type	not present	fertile, wild type phenotype
S 905	wild type	not present	fertile, wild type phenotype
S 906	wild type	present	fertile, wild type phenotype
S 907	wild type	not present	fertile, wild type phenotype

S 908	wild type	not present	fertile, wild type phenotype
S 909	wild type	not present	fertile, wild type phenotype
S 910	wild type	present	fertile, wild type phenotype
S 911	wild type	present	fertile, wild type phenotype
S 912	wild type	present	fertile, wild type phenotype
S 913	wild type	not present	fertile, wild type phenotype
S 918	wild type	not present	fertile, wild type phenotype
S 919	wild type	present	fertile, wild type phenotype
S 920	wild type	not present	fertile, wild type phenotype
S 922	wild type	not present	fertile, wild type phenotype
S 923	wild type	present	fertile, wild type phenotype
S 924	wild type	not present	fertile, wild type phenotype
S 925	wild type	present	fertile, wild type phenotype
S 926	wild type	not present	fertile, wild type phenotype
S 937	wild type	not present	fertile, wild type phenotype
S 938	wild type	not present	fertile, wild type phenotype
S 939	wild type	present	fertile, wild type phenotype
S 940	wild type	not present	fertile, wild type phenotype
S 941	wild type	present	fertile, wild type phenotype
S 942	wild type	not present	fertile, wild type phenotype
S 943	wild type	not present	fertile, wild type phenotype
S 944	wild type	not present	fertile, wild type phenotype
S 945	wild type	not present	fertile, wild type phenotype
S 946	wild type	not present	fertile, wild type phenotype
S 947	wild type	not present	fertile, wild type phenotype
S 948	wild type	present	fertile, wild type phenotype
S 949	wild type	present	fertile, wild type phenotype
S 950	wild type	not present	fertile, wild type phenotype
S 951	wild type	not present	fertile, wild type phenotype
S 952	wild type	present	fertile, wild type phenotype
S 953	wild type	not present	fertile, wild type phenotype
S 954	wild type	not present	fertile, wild type phenotype
S 955	wild type	not present	fertile, wild type phenotype
S 956	wild type	not present	fertile, wild type phenotype
S 957	wild type	not present	fertile, wild type phenotype
S 958	wild type	not present	fertile, wild type phenotype
S 963	wild type	present	fertile, wild type phenotype
S 28	Δasf1	present	sterile, Δasf1 phenotype
S 40	Δasf1	present	sterile, Δasf1 phenotype
S 49	Δasf1	present	sterile, Δasf1 phenotype
S 50	Δasf1	present	sterile, Δasf1 phenotype
S 54	Δasf1	present	sterile, Δasf1 phenotype
S 57	Δasf1	present	sterile, Δasf1 phenotype
S 691	Δasf1	present	sterile, Δasf1 phenotype
S 702	Δasf1	present	sterile, Δasf1 phenotype
S 790	Δasf1	present	sterile, Δasf1 phenotype
S 807	Δasf1	present	sterile, Δasf1 phenotype
S 914	Δasf1	present	sterile, Δasf1 phenotype
S 915	Δasf1	present	sterile, Δasf1 phenotype
S 916	Δasf1	present	sterile, Δasf1 phenotype

S 917	Δasf1	present	sterile, Δasf1 phenotype
S 921	Δasf1	present	sterile, Δasf1 phenotype
S 927	Δasf1	present	sterile, Δasf1 phenotype
S 928	Δasf1	present	sterile, Δasf1 phenotype
S 929	Δasf1	present	sterile, Δasf1 phenotype
S 930	Δasf1	present	sterile, Δasf1 phenotype
S 931	Δasf1	present	sterile, Δasf1 phenotype
S 932	Δasf1	present	sterile, Δasf1 phenotype
S 933	Δasf1	present	sterile, Δasf1 phenotype
S 934	Δasf1	present	sterile, Δasf1 phenotype
S 935	Δasf1	present	sterile, Δasf1 phenotype
S 936	Δasf1	present	sterile, Δasf1 phenotype
S 959	Δasf1	present	sterile, Δasf1 phenotype
S 960	Δasf1	present	sterile, Δasf1 phenotype
S 961	Δasf1	present	sterile, Δasf1 phenotype
S 962	Δasf1	present	sterile, Δasf1 phenotype
S 964	Δasf1	present	sterile, Δasf1 phenotype
S 980	Δasf1	present	sterile, Δasf1 phenotype
S 981	Δasf1	present	sterile, Δasf1 phenotype
S 993	Δasf1	present	sterile, Δasf1 phenotype
S 1015	Δasf1	present	sterile, Δasf1 phenotype
S 1019	Δasf1	present	sterile, Δasf1 phenotype
S 1023	Δasf1	present	sterile, Δasf1 phenotype
S 1025	Δasf1	present	sterile, Δasf1 phenotype
J 3	Δasf1	present	sterile, Δasf1 phenotype
J 14	Δasf1	present	sterile, Δasf1 phenotype
J 37	Δasf1	present	sterile, Δasf1 phenotype
J 71	Δasf1	present	sterile, Δasf1 phenotype
J 78	Δasf1	present	sterile, Δasf1 phenotype
SJM 22.1.3	Δasf1 + pDS23-ASF1-WT	present	fertile, wild type phenotype
SJM 25.4.2	Δasf1 + pDS23-ASF1-WT	present	fertile, wild type phenotype
SJM 25.2.2	Δasf1 + pDS23-ASF1-WT	not present	fertile, wild type phenotype
SJM 25.2.4	Δasf1 + pDS23-ASF1-WT	not present	fertile, wild type phenotype
SJM 26.8.1	Δasf1 + pDS23-ASF1-D37A	not present	fertile, growth aberrations
SJM 26.8.4	Δasf1 + pDS23-ASF1-D37A	not present	fertile, growth aberrations
SJM 26.8.5	Δasf1 + pDS23-ASF1-D37A	not present	fertile, growth aberrations
SJM 26.8.8	Δasf1 + pDS23-ASF1-D37A	not present	fertile, growth aberrations
SJM 26.8.9	Δasf1 + pDS23-ASF1-D37A	present	fertile, growth aberrations
SJM 26.8.11	Δasf1 + pDS23-ASF1-D37A	not present	fertile, growth aberrations
SJM 26.8.12	Δasf1 + pDS23-ASF1-D37A	present	fertile, growth aberrations

SJM 27.4.1	Δasf1 + pDS23-ASF1-V94R	not present	sterile, Δasf1 phenotype
JB 13.1.1	Δasf1 + pDS23-ASF1-V94R	not present	sterile, Δasf1 phenotype
JB 13.1.2	Δasf1 + pDS23-ASF1-V94R	not present	sterile, Δasf1 phenotype
JB 13.1.3	Δasf1 + pDS23-ASF1-V94R	present	sterile, Δasf1 phenotype
JB 13.1.4	Δasf1 + pDS23-ASF1-V94R	present	sterile, Δasf1 phenotype
JB 13.1.5	Δasf1 + pDS23-ASF1-V94R	present	sterile, Δasf1 phenotype
JB 13.1.6	Δasf1 + pDS23-ASF1-V94R	not present	sterile, Δasf1 phenotype
JB 13.1.7	Δasf1 + pDS23-ASF1-V94R	not present	sterile, Δasf1 phenotype
JB 13.1.8	Δasf1 + pDS23-ASF1-V94R	not present	sterile, Δasf1 phenotype
JB 13.1.9	Δasf1 + pDS23-ASF1-V94R	not present	sterile, Δasf1 phenotype
JB 13.1.10	Δasf1 + pDS23-ASF1-V94R	not present	sterile, Δasf1 phenotype
JB 13.1.11	Δasf1 + pDS23-ASF1-V94R	present	sterile, Δasf1 phenotype
JB 13.1.12	Δasf1 + pDS23-ASF1-V94R	present	sterile, Δasf1 phenotype
JB 13.1.13	Δasf1 + pDS23-ASF1-V94R	not present	sterile, Δasf1 phenotype
JB 13.1.114	Δasf1 + pDS23-ASF1-V94R	not present	sterile, Δasf1 phenotype
JB 41.1.1	Δasf1 + pGG-ASF1-152T	not present	sterile, Δasf1 phenotype
JB 41.1.3	Δasf1 + pGG-ASF1-152T	not present	sterile, Δasf1 phenotype
JB 41.1.18	Δasf1 + pGG-ASF1-152T	present	sterile, Δasf1 phenotype
JB 41.1.22	Δasf1 + pGG-ASF1-152T	not present	sterile, Δasf1 phenotype
JB 41.4.3	Δasf1 + pGG-ASF1-152T	present	sterile, Δasf1 phenotype
JB 42.1.2	Δasf1 + pGG-ASF1-185T	present	sterile, Δasf1 phenotype
JB 42.1.3	Δasf1 + pGG-ASF1-185T	present	sterile, Δasf1 phenotype
JB 42.2.1	Δasf1 + pGG-ASF1-185T	not present	sterile, Δasf1 phenotype
JB 42.2.2	Δasf1 + pGG-ASF1-185T	not present	sterile, Δasf1 phenotype
JB 42.3.1	Δasf1 + pGG-ASF1-185T	not present	sterile, Δasf1 phenotype
JB 42.4.1	Δasf1 + pGG-ASF1-185T	not present	sterile, Δasf1 phenotype
JB 42.4.3	Δasf1 + pGG-ASF1-185T	not present	sterile, Δasf1 phenotype
JB 42.5.1	Δasf1 + pGG-ASF1-185T	not present	sterile, Δasf1 phenotype
JB 42.6.1	Δasf1 + pGG-ASF1-185T	not present	sterile, Δasf1 phenotype
JB 51.1.13	Δasf1 + pGG-ASF1-210T	present	fertile, wild type phenotype
JB 51.1.18	Δasf1 + pGG-ASF1-210T	present	fertile, wild type phenotype
JB 51.4.1	Δasf1 + pGG-ASF1-210T	present	fertile, wild type phenotype

A

genome version	**v04**	**v03**
genome size [Mb]	39.4	38.9
N50 [kb]	5713	180
no. of contigs/scaffolds	7	584
no. of gaps in contigs/scaffolds	0	216
largest contig [kb]	9310	664
smallest contig [kb]	4093	1
GC content [%]	52.1	52.0
no. of protein-coding genes	10420	9874

B

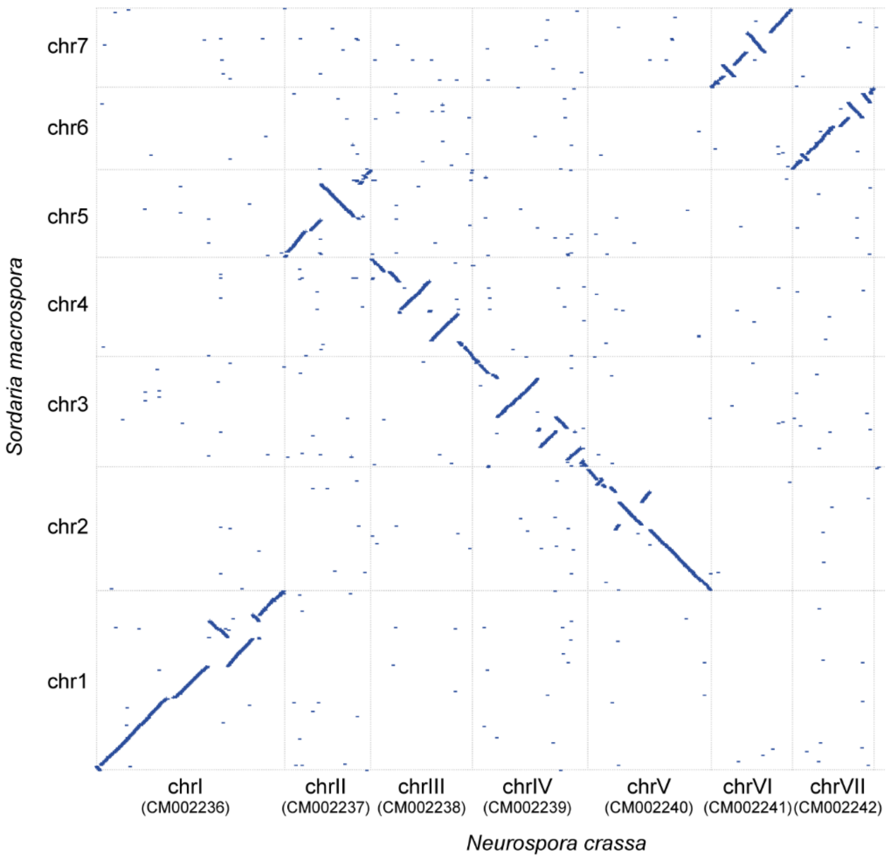

Supplemental Figure 1. Features of the *Sordaria macrospora* genome assembly v04. A. Core features of the S. macrospora assembly versions v04 (this study) and v03 (Blank-Landeshammer et al. 2019, mBio 10:e02367-02319). B. Nucmer (Kurtze et al. 2004, Genome Biol 5:R12) comparison of the genome assemblies of *S. macrospora* and its close relative *Neurospora crassa* (FungiDB version 52; Galagan et al. 2003, Nature 422:859-868; Basenko et al. 2018, J Fungi 4:39). The nuclear genomes of both ascomycetes comprise seven chromosomes that are largely syntenic except for several inversions or shuffling of regions within each chromosome.

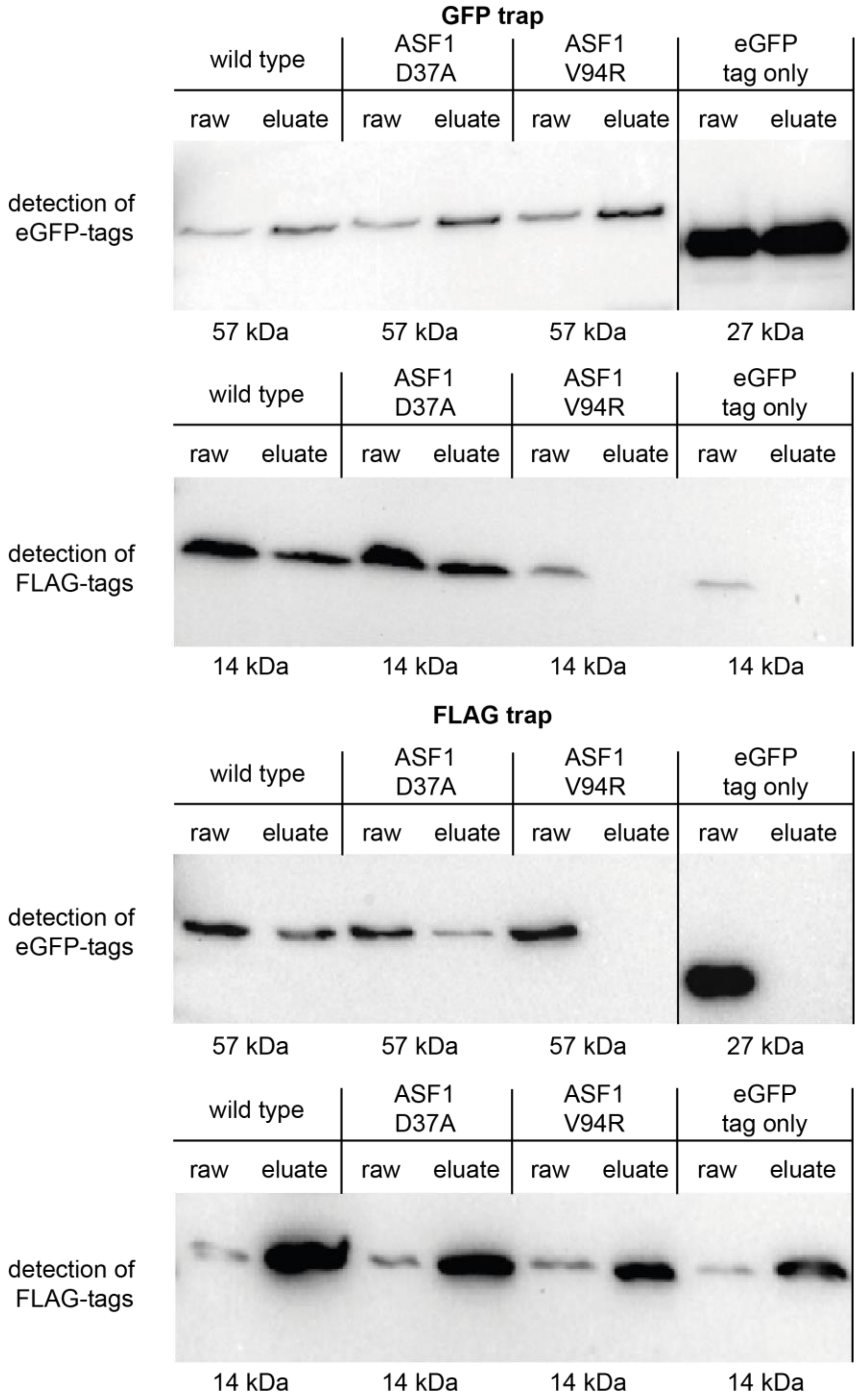

Supplemental Figure 2. Co-immunoprecipitation results for ASF1 variants with amino acid substitutions and histone H4. GFP-tagged ASF1 wild type and variants D37A and V94R were used as potential interaction partners for Flag-tagged histone H4 in a GFP trap and a Flag trap. Results were checked by western blot analysis with antibodies against GFP and Flag tags. ASF1 wild type and the D37A variant showed signals for bait and prey proteins in the raw and eluate sample, indicating interaction, whereas the V94R variant showed the signal for the prey protein only in the raw sample. Strains expressing non-fused GFP with the corresponding Flag-tagged H4 were used as a negative control. Uncropped blots are shown in Supplementary Figure 3B.

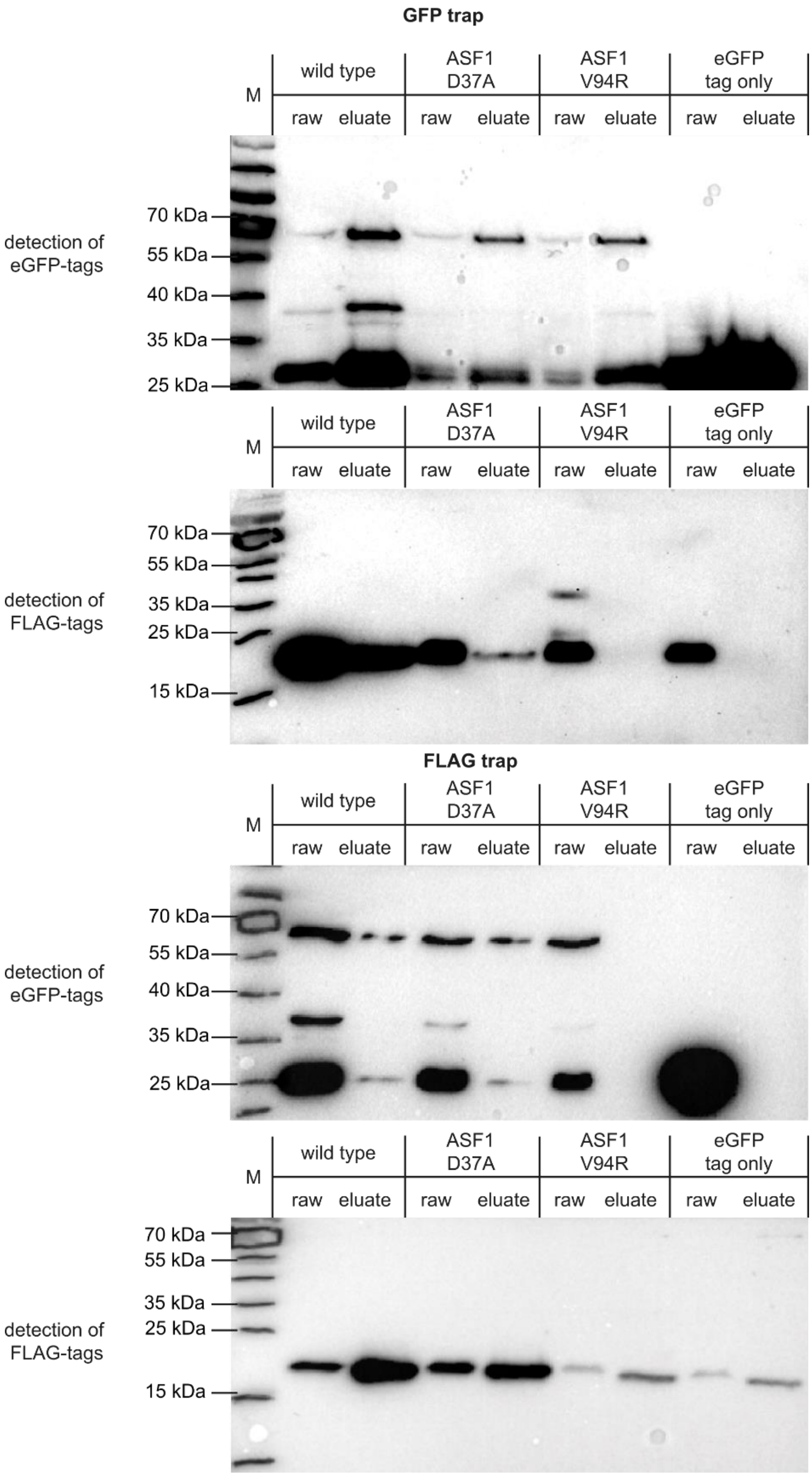

Supplemental Figure 3A. Uncropped blots - Co-immunoprecipitation results for ASF1 variants with amino acid substitutions and histone H3. GFP-tagged ASF1 wild type and variants D37A and V94R were used as potential interaction partners for Flag-tagged histone H3 in a GFP trap and a Flag trap. Results were checked by western blot analysis with antibodies against GFP and Flag tags. ASF1 wild type and the D37A variant showed signals for bait and prey proteins in the raw and eluate sample, indicating interaction, whereas the V94R variant showed the signal for the prey protein only in the raw sample. Strains expressing non-fused GFP with the corresponding Flag-tagged H3 were used as a negative control.

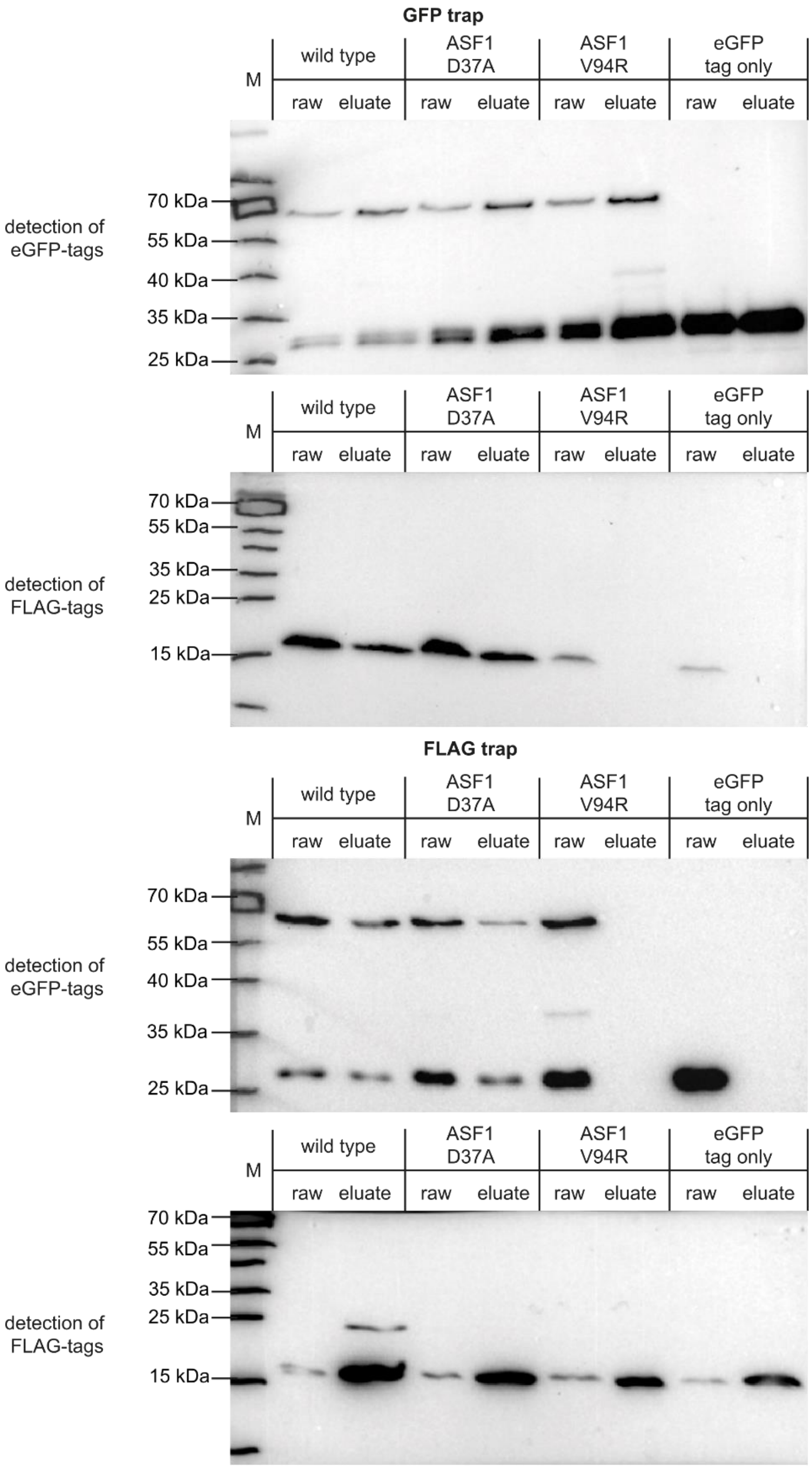

Supplemental Figure 3B. Uncropped blots - Co-immunoprecipitation results for ASF1 variants with amino acid substitutions and histone H4. GFP-tagged ASF1 wild type and variants D37A and V94R were used as potential interaction partners for Flag-tagged histone H4 in a GFP trap and a Flag trap. Results were checked by western blot analysis with antibodies against GFP and Flag tags. ASF1 wild type and the D37A variant showed signals for bait and prey proteins in the raw and eluate sample, indicating interaction, whereas the V94R variant showed the signal for the prey protein only in the raw sample. Strains expressing non-fused GFP with the corresponding Flag-tagged H4 were used as a negative control.

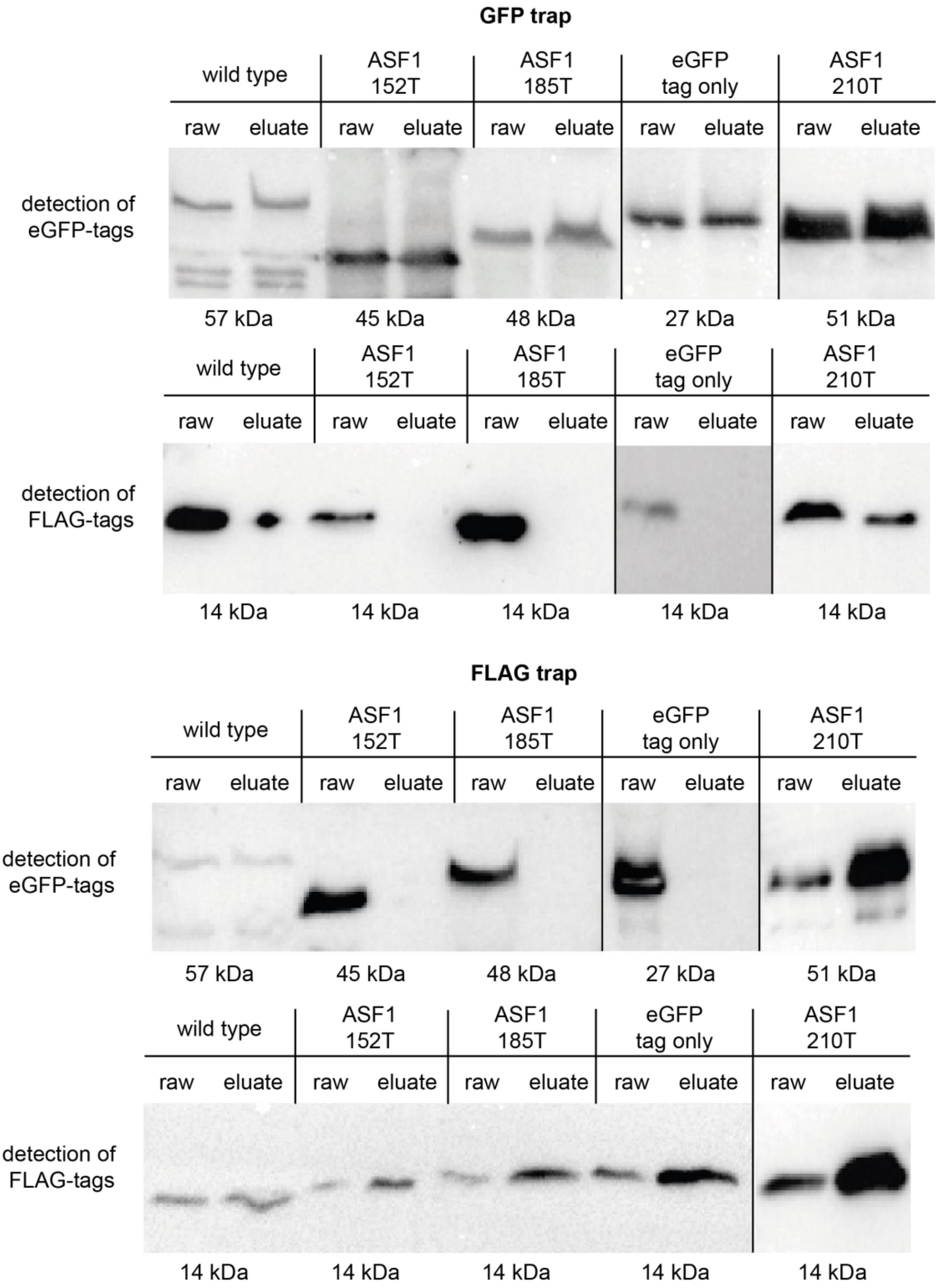

Supplemental Figure 4. Co-immunoprecipitation results for truncated ASF1 variants and histone H4. GFP-tagged ASF1 wild type and variants 152T, 185T and 210T were used as potential interaction partners for Flag-tagged histone H4 in a GFP trap and a Flag trap. Results were checked by western blot analysis with antibodies against GFP and Flag tags. ASF1 wild type and the 210T variant showed signals for bait and prey proteins in the raw and eluate sample, indicating interaction, whereas the 152T and 185T variants showed the signal for the prey protein only in the raw sample. Strains expressing non-fused GFP with the corresponding Flag-tagged H4 were used as a negative control. Uncropped blots are shown in Supplemental Figure 5B.

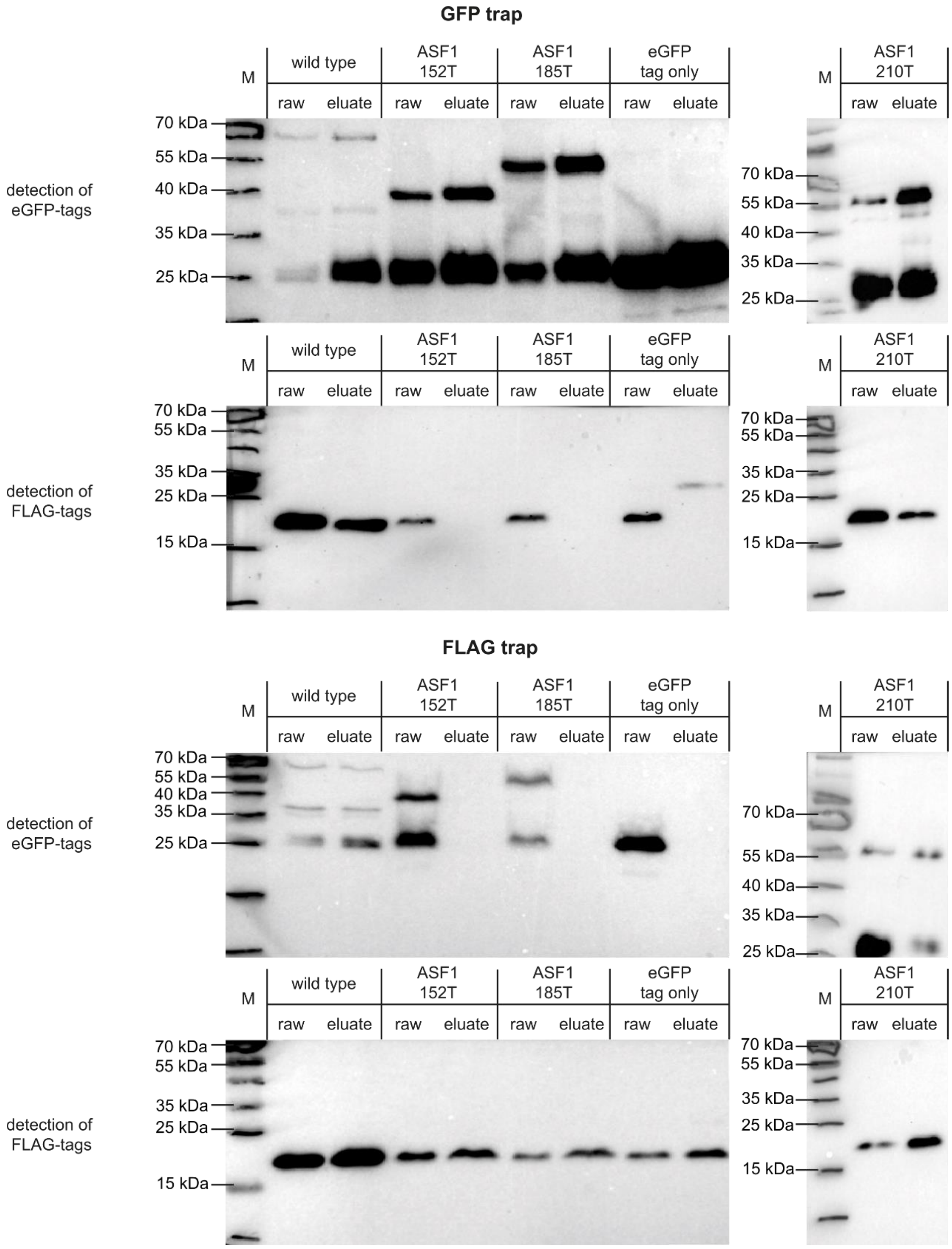

Supplemental Figure 5A. Uncropped blots - Co-immunoprecipitation results for truncated ASF1 variants and histone H3. GFP-tagged ASF1 wild type and variants 152T, 185T and 210T were used as potential interaction partners for Flag-tagged histone H3 in a GFP trap and a Flag trap. Results were checked by western blot analysis with antibodies against GFP and Flag tags. ASF1 wild type and the 210T variant showed signals for bait and prey proteins in the raw and eluate sample, indicating interaction, whereas the 152T and 185T variants showed the signal for the prey protein only in the raw sample. Strains expressing non-fused GFP with the corresponding Flag-tagged H3 were used as a negative control.

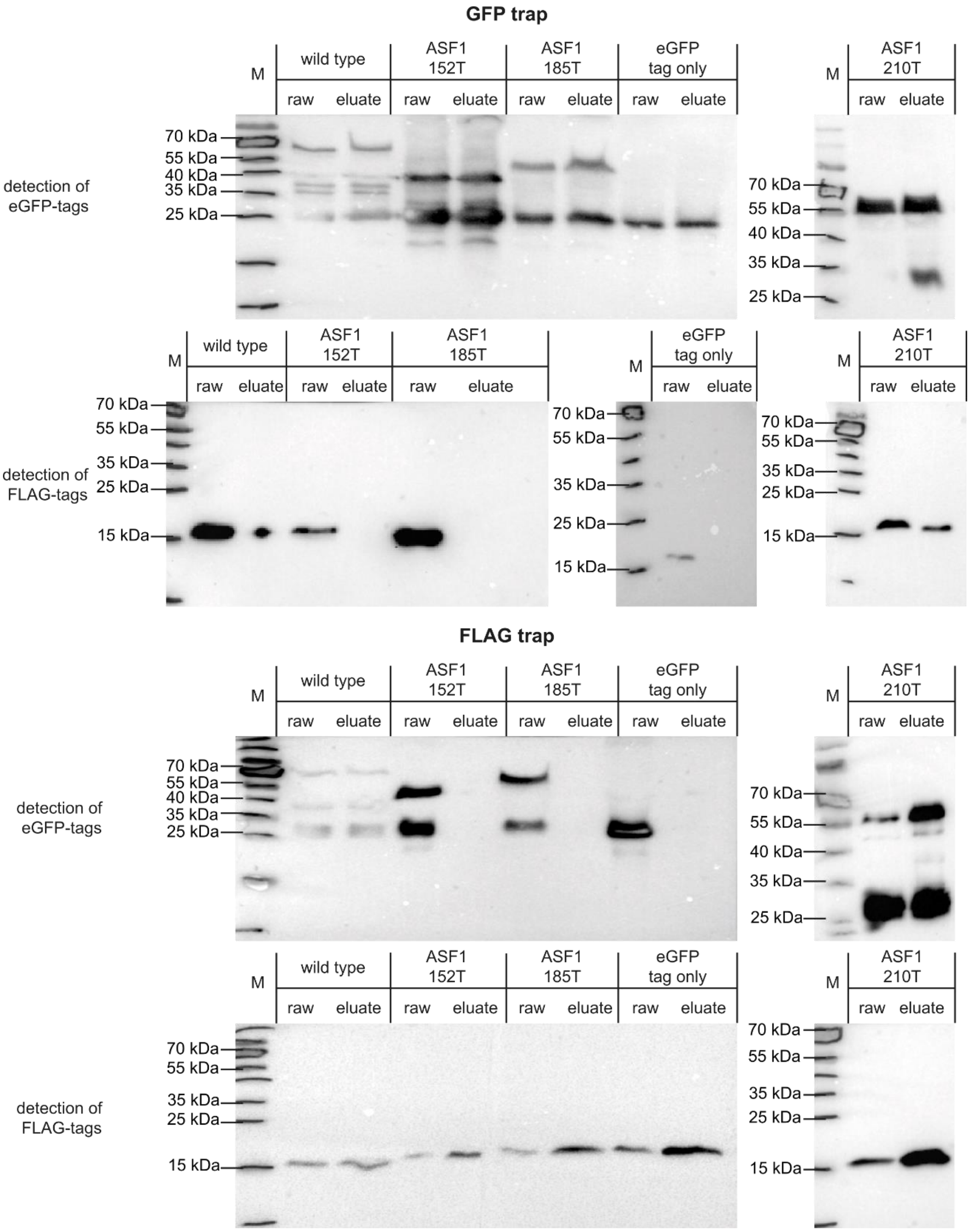

Supplemental Figure 5B. Uncropped blots - Co-immunoprecipitation results for truncated ASF1 variants and histone H4. GFP-tagged ASF1 wild type and variants 152T, 185T and 210T were used as potential interaction partners for Flag-tagged histone H4 in a GFP trap and a Flag trap. Results were checked by western blot analysis with antibodies against GFP and Flag tags. ASF1 wild type and the 210T variant showed signals for bait and prey proteins in the raw and eluate sample, indicating interaction, whereas the 152T and 185T variants showed the signal for the prey protein only in the raw sample. Strains expressing non-fused GFP with the corresponding Flag-tagged H4 were used as a negative control.

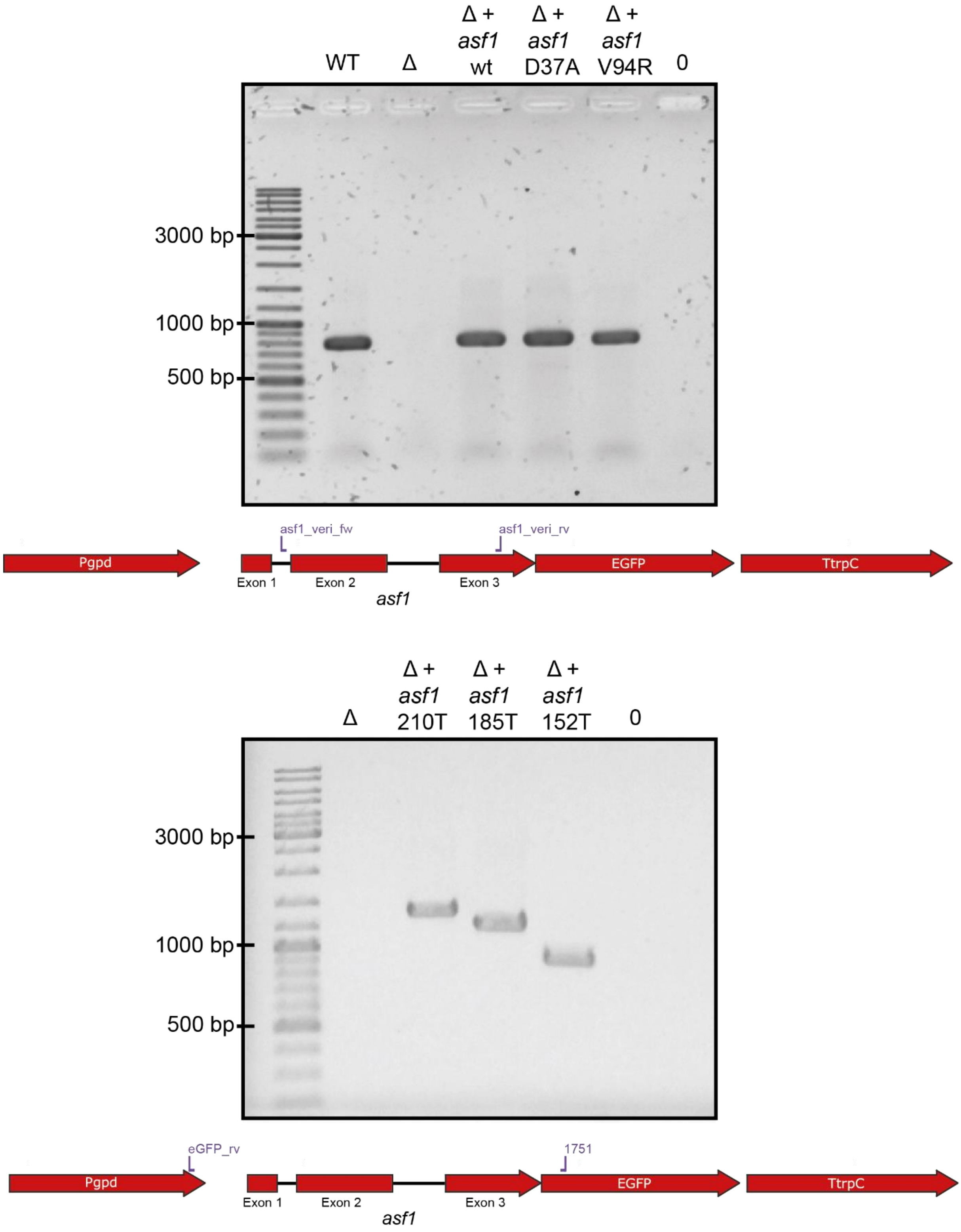

Supplemental Figure 6. PCR verification of transformation of Δasf1 with *asf1* variants. Primers asf1_veri_fw and asf1_very_rv were used to confirm the integration of the *asf1* wild type, D37A and V94R constructs, amplifying a 786 bp region within the *asf1* gene, which is not present in the deletion mutant. Primers 1751 and eGFP_rv were used to amplify parts of the integrated complementation vectors for *asf1* 152T, 185T and 210T variants, allowing to also verify the integration of the correct length of the truncated variants. The expected bands of 1282 bp for *asf1* 210T, 1207 bp for *asf1* 185T and 918 bp for *asf1* 152T were detected. Abbreviation: Δ = Δasf1, 0 = no template control.

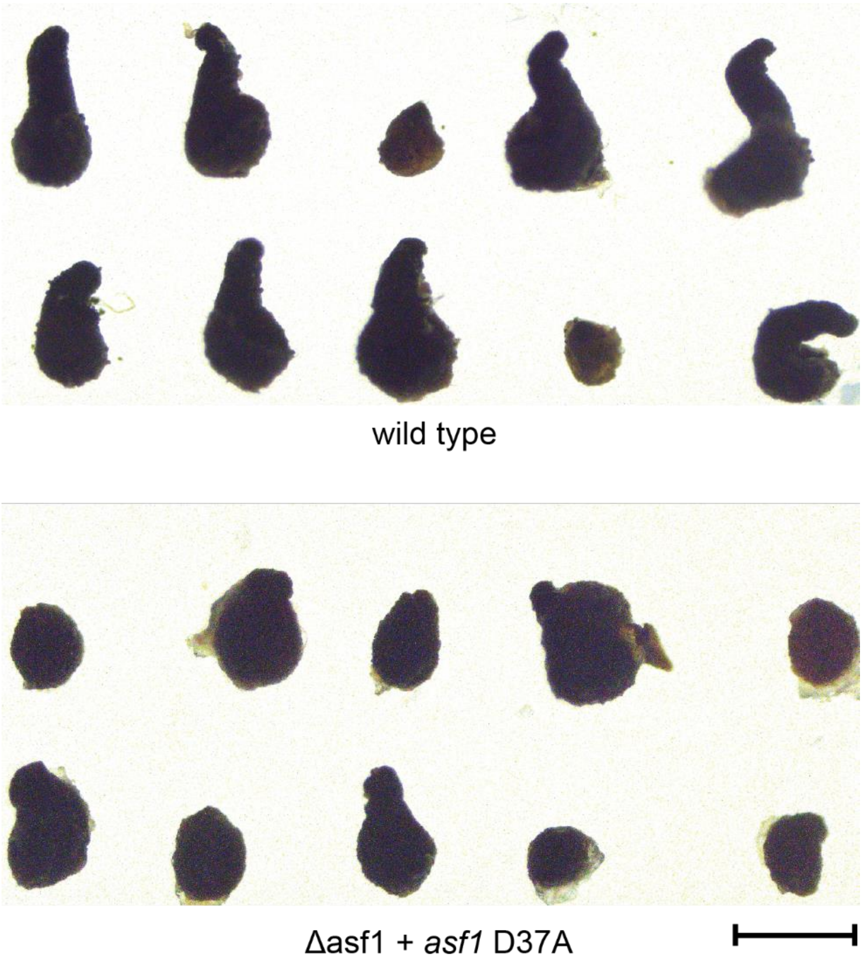

Supplemental Figure 7. Comparison of fruiting body morphology of wild type and Δasf1 + asf1 D37A. While the wild type produces fully grown, cone-shaped perithecia as well as protoperithecia, strains expressing asf1 with a D37A substitution tend to produce perithecia that appear more rounded and look like giant protoperithecia, although the production of normal perithecia is generally possible. The scale bar represents 300 µm.

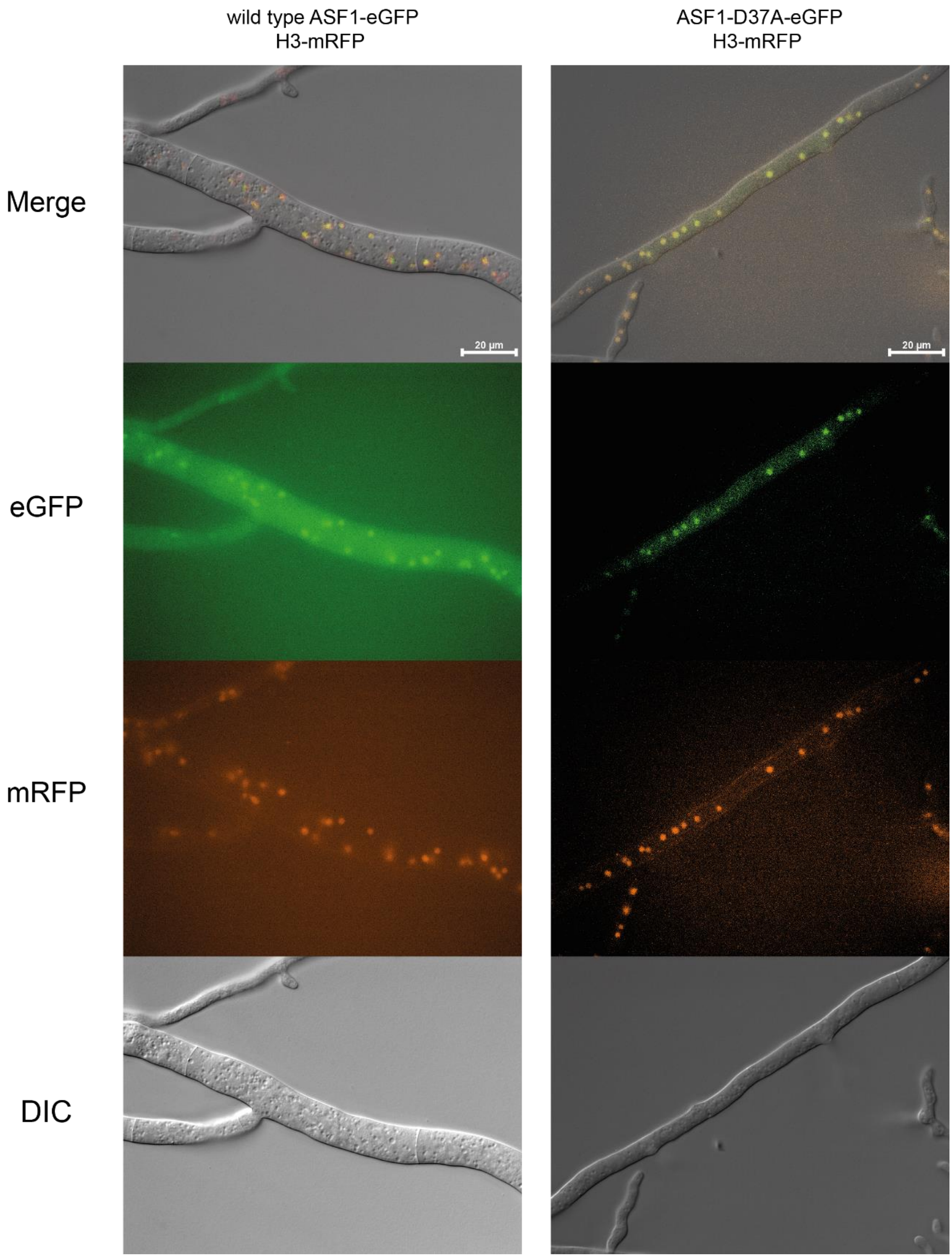

Supplemental Figure 8A. Localization of ASF1-variants by fluorescence microscopy. To confirm the correct localization of ASF1 variants in the nucleus, eGFP tagged variants were grown together with a control strain expressing histone H3 with an mRFP tag. Hyphal fusion of both strains leads to nuclear exchange and therefore hyphae that contain both tagged proteins. Both tags colocalized, detectable in the shown overlay images (GFP fluorescence, mRFP fluorescence and differential interference contrast to show hyphal outlines) by yellow - orange fluorescence, thus confirming the nuclear localization of all ASF1 variants.

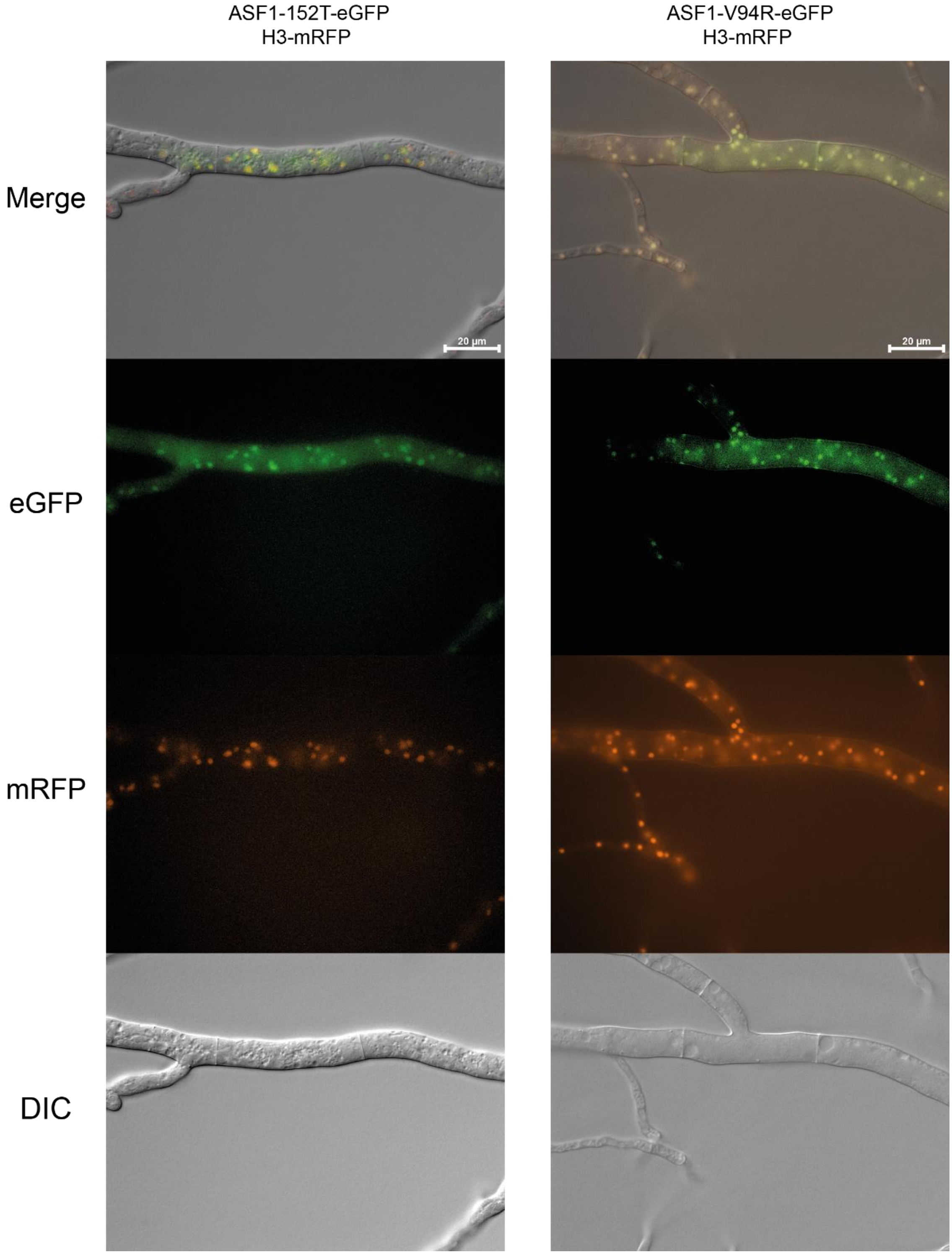

Supplemental Figure 8B. Localization of ASF1-variants by fluorescence microscopy. To confirm the correct localization of ASF1 variants in the nucleus, eGFP tagged variants were grown together with a control strain expressing histone H3 with an mRFP tag. Hyphal fusion of both strains leads to nuclear exchange and therefore hyphae that contain both tagged proteins. Both tags colocalized, detectable in the shown overlay images (GFP fluorescence, mRFP fluorescence and differential interference contrast to show hyphal outlines) by yellow - orange fluorescence, thus confirming the nuclear localization of all ASF1 variants.

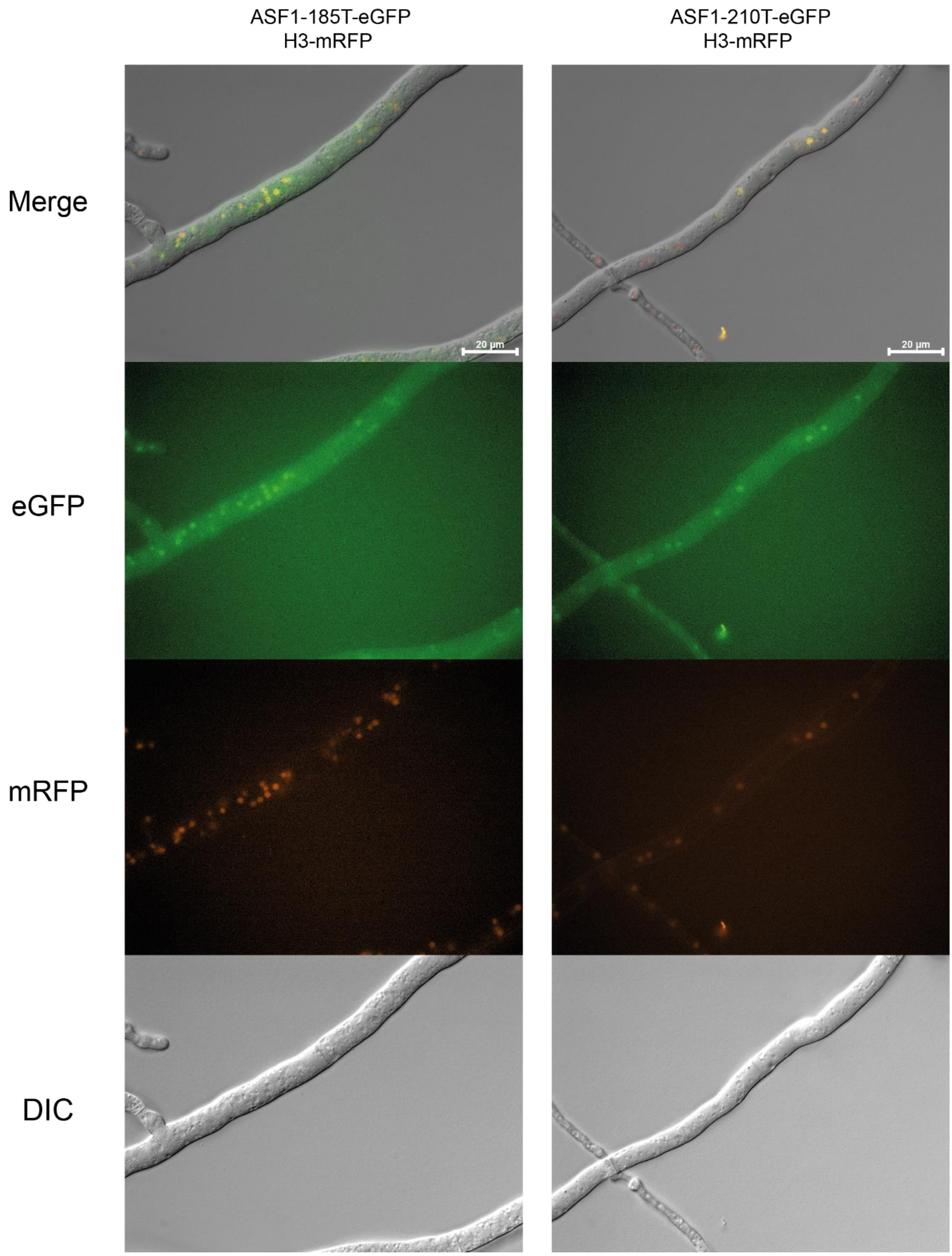

Supplemental Figure 8C. Localization of ASF1-variants by fluorescence microscopy. To confirm the correct localization of ASF1 variants in the nucleus, eGFP tagged variants were grown together with a control strain expressing histone H3 with an mRFP tag. Hyphal fusion of both strains leads to nuclear exchange and therefore hyphae that contain both tagged proteins. Both tags colocalized, detectable in the shown overlay images (GFP fluorescence, mRFP fluorescence and differential interference contrast to show hyphal outlines) by yellow - orange fluorescence, thus confirming the nuclear localization of all ASF1 variants.

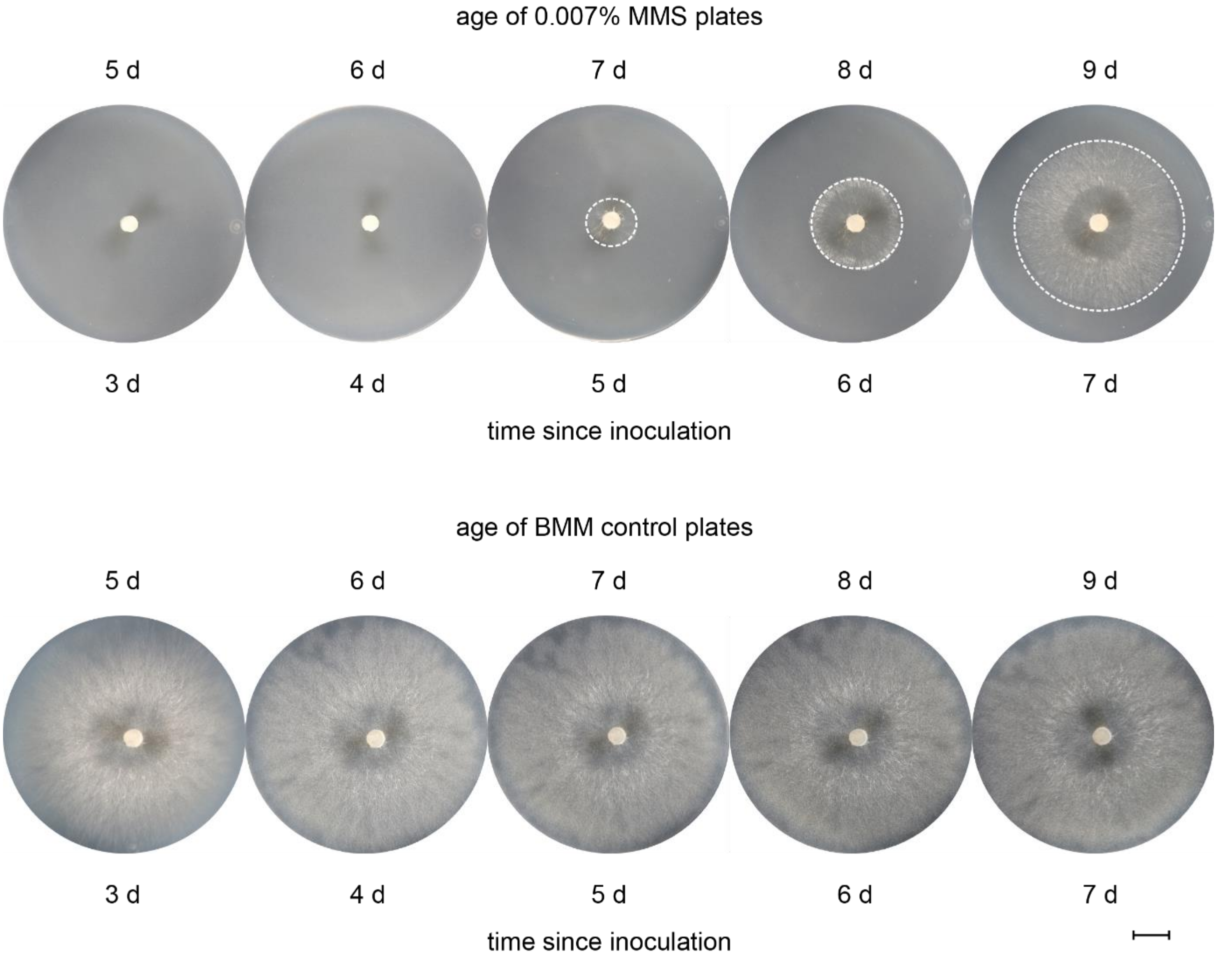

Supplemental Figure 9. Validation of the MMS assay. To validate the effect of the highly unstable substance MMS, the sensitive strain Δasf1 was inoculated on 5-day-old BMM plates with 0.007% MMS. While no growth was possible until day 6, small amounts of mycelia were visible by day 7. Thereafter, the strain appeared to be able to grow on the media. Freshly prepared BMM medium containing 0.007% MMS is therefore suitable for the for the 4-day observation period used in the genotoxic stress assay.

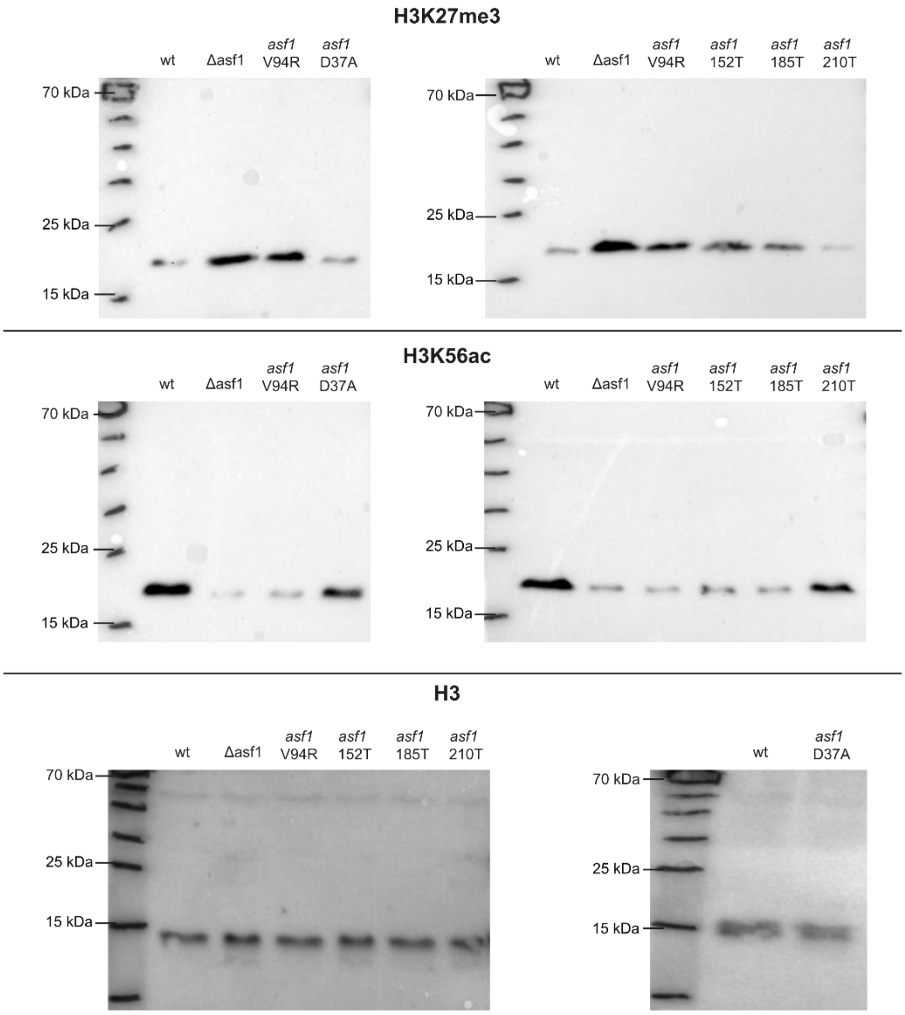

Supplemental Figure 10. Uncropped blots - Semiquantitative screening for histone modifications affected by ASF1. Western blots with antibodies against the indicated histone modifications were used to compare the band strength of equal amounts of protein from wild type, Δasf1 and strains expressing *asf1* variants. H3 levels were measured as an internal control.

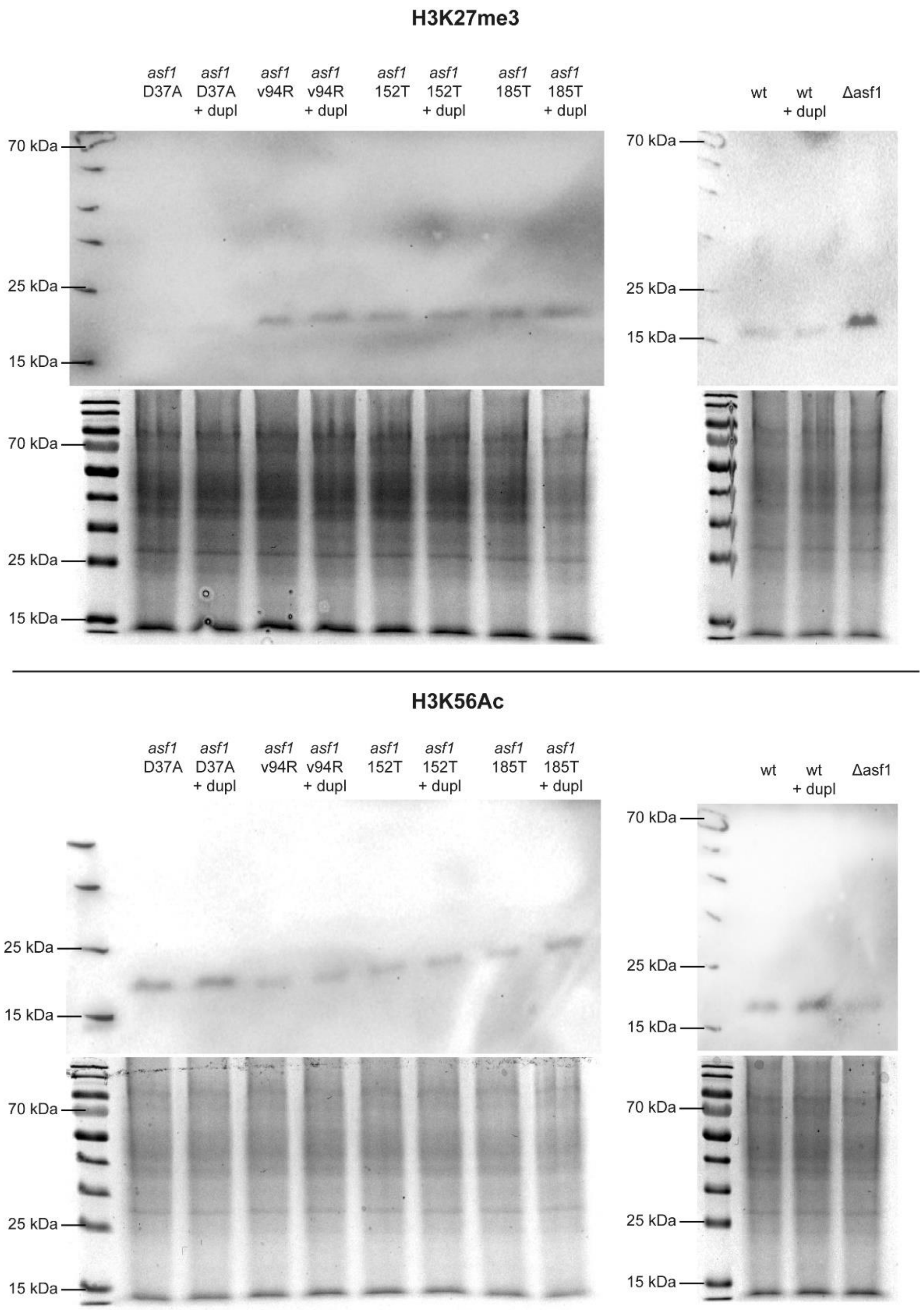

Supplemental Figure 11. Histone modification levels in strains with or without duplication on chromosome 2. Western blots with antibodies against the indicated histone modifications were used to compare the band strength of equal amounts of protein from strains expressing ASF1 variants. To assess a possible effect of the duplication on chromosome 2 detected in the Hi-C experiments, strains with and without the duplication were used. Coomassie gels were used to detect possible differences in total protein amount. Strain numbers: Wild type - duplication = SN1693; wild type + duplication = S710; *asf1* V94R - duplication = SJM 27.4.1; *asf1* V94R + duplication = JB 13.1.4; *asf1* D37A - duplication = SJM 26.8.5; *asf1* D37A + duplication = SJM 26.8.9; *asf1* 152T - duplication = JB 41.1.1; *asf1* 152T + duplication = JB 41.1.18; *asf1* 185T - duplication = JB 42.2.2; *asf1* 185T + duplication = JB 42.1.2
